# Simultaneous Burr hole drainage and middle meningeal artery occlusion through a single incision for chronic subdural hematoma guided by 3D reconstruction

**DOI:** 10.3389/fneur.2026.1852386

**Published:** 2026-07-14

**Authors:** Sen He, Taise Mosso Ramos, Fang Xue, Wenyan Zhang, Fei Xie

**Affiliations:** 1 Department of Neurosurgery, West China Hospital of Sichuan University-Ziyang Hospital (Ziyang Central Hospital), Ziyang, China; 2 Department of Neurosurgery, Agostinho Neto Central Hospital, Praia, Cape Verde

**Keywords:** Burr hole drainage, chronic subdural hematoma, improved technology, middle meningeal artery, recurrence, three-dimensional reconstruction

## Abstract

**Background:**

Burr hole drainage (BHD) is the preferred surgical technique for chronic subdural hematoma (CSDH), but the high recurrence rate of hematoma after surgery remains a clinical challenge. To minimize postoperative hematoma recurrence, this study aimed to investigate the clinical outcomes of performing CSDH drainage and middle meningeal artery (MMA) occlusion through the same cranial burr hole.

**Methods:**

Patients with CSDH from January 2021 to June 2025 were collected, and divided into an observation group and a control group based on the surgical method implemented. A single-point cranial burr hole was determined through three-dimensional (3D) reconstruction technology to locate the MMA in the observation group, then hematoma was drained and MMA was blocked through same cranial burr hole. The control group underwent traditional BHD procedures. Both groups received the same conventional nursing and postoperative treatment.

**Results:**

There were 41 patients in the observation group and 75 in the control group. The demographic characteristics, medical history, mRS score at admission, hematoma thickness, and degree of midline shift were comparable between both groups at enrollment. Both groups showed no significant differences in intraoperative indicators, hematoma removal effect, perioperative complications, hospitalization costs or mRS score at 6 months postoperatively. The observation group experienced shorter drainage duration and hospitalization duration (*p* < 0.001), and had less hematoma residual volume at 1 month postoperatively (*p* < 0.001) compared to the control group. In addition, the hematoma recurrence rate at 6 months postoperatively was significantly lower in the observation group than in the control group (4.9% vs. 20.0%, *p* = 0.030).

**Conclusion:**

This surgical approach, which involved BHD and MMA through a single point under 3D reconstruction technique, led to better drainage of hematoma for CSDH patients compared to the conventional method. It contributed to shorter surgical and hospitalization duration, reduced hematoma residual volume, and a lower risk of hematoma recurrence after surgery.

## Introduction

The incidence rate of chronic subdural hemorrhage (CSDH) continues to rise along with long-term mortality, especially in the aging population with increased use of antithrombotic drugs (1–3). Burr hole drainage (BHD) has become a common surgical procedure for patients with CSDH due to its advantages such as safety, minimal damage, and immediate relief of space-occupying effect (4). However, postoperative recurrence rate of hematoma remains the challenge, which has been reported to range from 10 to 30% (5, 6). The hematoma recurrence not only increases the medical and economic burden but also severely affects the patient’s quality of life (5, 6).

Currently, it is believed that the local inflammatory cascade reaction triggered by mild head trauma, as well as the formation and rupture of new blood vessels in the outer membrane are significant factors contributing to the formation and recurrence of CSDH (7, 8). Based on these theories, the middle meningeal artery embolization(MMAE) is widely explored in patients with CSDH as a minimally invasive treatment method (7–9). However, MMAE cannot immediately relieve the occupying effect of hematoma, and is limited in application for its expensive interventional equipment (10, 11). To address this contradiction, some researchers have explored directly treating MMA during BHD surgery, aiming to combine the advantages of both therapies. Preliminary studies suggest that simultaneously draining the hematoma and occluding the MMA may reduce the risk of postoperative recurrence (11–14). However, the existing methods mostly rely on specialized equipment such as intraoperative navigation, microscopes, 3D printing technology, or specially designed positioning guides (11–14). In addition, bilateral BHD is commonly used in previous studies (15, 16). However, there is no literature reporting the use of 3D reconstruction technology to locate the MMA during BHD in patients with CSDH through a single-point hole.

This study retrospectively analyzed the data of patients with CSDH between conventional BHD and single-point expanded BHD combined with MMA occlusion under the guiding of 3D reconstruction. It explored the effect of this improved technique in hematoma clearance and postoperative hematoma recurrence reduction in CSDH patients.

## Materials and methods

### Patients

The study protocol has been approved by the Ethics Committee of Ziyang Hospital of West China Hospital, Sichuan University (approval number: 2024199). The written informed consent was obtained from each participant.

This study retrospectively analyzed the clinical data of 116 patients with CSDH who underwent surgical treatment from January 2021 to June 2025 in the Department of Neurosurgery at Ziyang Hospital of West China Hospital, Sichuan University.

Inclusion criteria were as follows: (1) Patients aged 18 or older; (2) Diagnosed with CSDH by cranial CT or MRI, accompanied by obvious clinical symptoms such as headache, nausea, limb weakness, and numbness; (3) Imaging indicating good liquefaction of the hematoma, uniform density, without septation or calcification; (4) Imaging showing a midline shift ≥0.5 cm; (5) Undergoing BHD under general anesthesia. Exclusion criteria were as follows: (1) Patients with comorbidities such as hematological diseases, autoimmune diseases, intracranial tumors, or cerebrovascular diseases such as Moyamoya disease or aneurysms; (2) Patients who had undergone craniotomy within the past year or have previously received craniotomy treatment on the same side of the hematoma; (3) Patients with CSDH who underwent neuronavigation, neuroendoscopy-assisted surgery, or interventional procedures; (4) Patients with bilateral hematomas who received surgical treatment; (5) Patients requiring hormone therapy due to underlying diseases; (6) Patients who used antithrombotic drugs within 1 month after surgery; (7) Patients with incomplete data.

### Grouping

The patients were classified into the observation group and the control group based on their actual surgical plans. A single cranial burr hole was determined through 3D reconstruction technology in the observation group, then hematoma was drained and MMA was blocked through the same cranial hole. The control group underwent traditional BHD procedures. Both groups received the same standard nursing procedures and postoperative treatment.

### Anesthesia procedures

All patients underwent endotracheal intubation and received general anesthesia from the same team of anesthesiologists. The vital signs and blood oxygen saturation of the patients were monitored continuously throughout the surgery. After the surgery, patients were resuscitated, and the endotracheal intubation was removed. The surgeries were all performed by senior neurosurgeons from the same medical team.

### Surgical incision design

**Figure 1:**
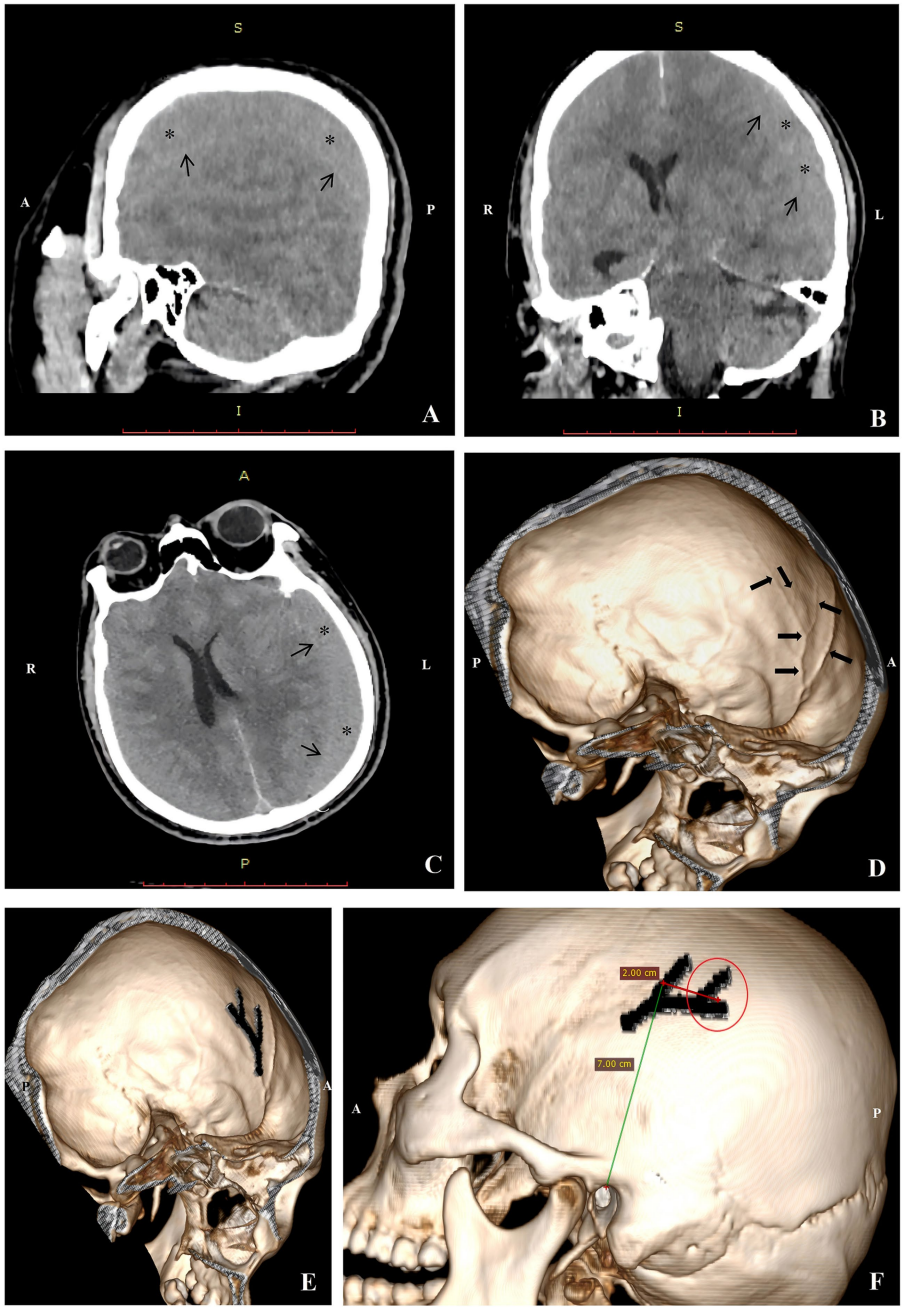
The surgical incision design method for patients in the observation group based on 3D reconstruction. **(A)** Sagittal CT scan demonstrating the distribution of the left chronic subdural hematoma (CSDH). **(B)** Coronal CT scan illustrating the distribution of the left CSDH. **(C)** Axial CT scan showing the distribution of the left CSDH. **(D)** Three-dimensional reconstruction highlighting the impression of the middle meningeal artery (MMA) on the inner table of the skull. **(E)** The silhouette function of the software was applied to remove the osseous impression of the MMA, revealing the underlying vascular course. **(F)** Using the external auditory canal as a reference point, the overlapping area between the MMA and the thicker region of the hematoma was marked, indicating the site for enlarged burr hole placement (red circle). The asterisk (*) in A-C denotes the CSDH; the thin arrow in A-C indicates the margin of the CSDH; the thick black arrow in **D** indicates the trajectory of the MMA. S, Superior; P, Posterior; A, Anterior; I, Inferior; R, Right; L, Left.

In the observation group, the incision was designed under 3D reconstruction. The DICOM data of the patient’s cranial CT, with a scan layer thickness of 5 mm or less, was imported into the RadiAnt DICOM Viewer (Medixant, Poznan, Poland). This software’s “3D Volume Rendering” function was used to perform 3D reconstruction of the data. The reconstruction revealed the lateral surface of the cranial cavity, showing the trajectory and impression of the major MMA within the skull after entering the cranium (Figures 1A–C). In the 3D VR interface, the Scalpel function was used to remove the hematoma on the opposite side of the skull. The internal side of the reconstructed cranial cavity was displayed, showing the path and pressure marks of the MMA as it entered the skull. The relationship between the main branches of the MMA and the hematoma was analyzed, and the trajectory of these main branches of the MMA on the dura mater located on the surface of the hematoma was identified (Figure 1D). The Scalpel function was used again to remove the impression trajectory of these vessels on the inner table of the skull (Figure 1E). The 3D Rotate function was then used to rotate the skull, with the external auditory canal marked as a reference point to help locate the path of MMA on the skull’s surface. A longitudinal surgical incision approximately 4 cm long was designed at the overlapping position of the aforementioned vessel trajectory and the thicker part of the hematoma (Figure 1F).

For patients in the control group, a longitudinal straight incision approximately 4 cm long was directly designed at the body surface projection of the thickest part of the hematoma.

### Surgical procedures

**Figure 2:**
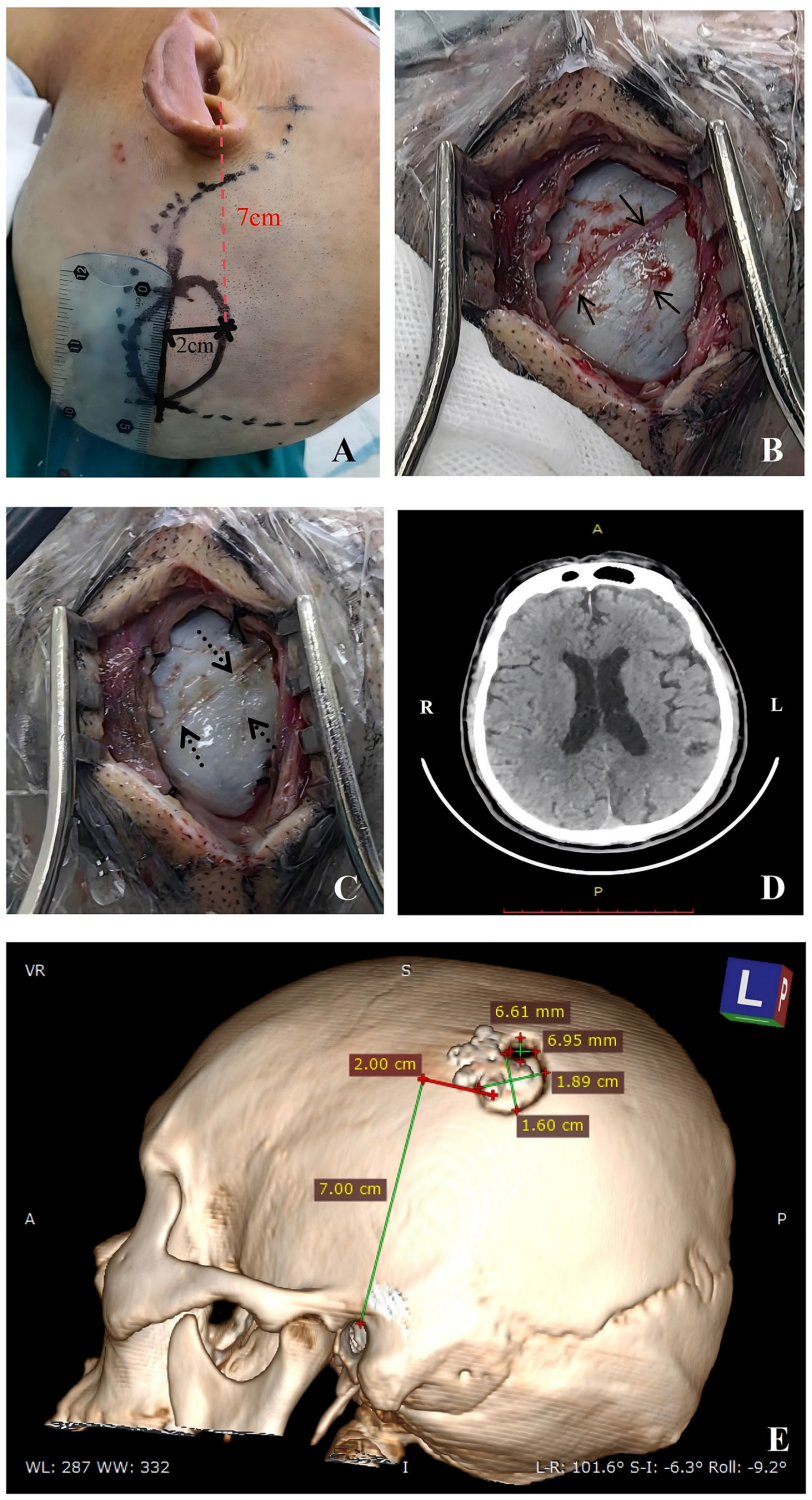
Surgical procedures in the observation group. **(A)** The surgical incision was designed based on three-dimensional reconstruction landmarks. **(B)** Following craniotomy, the middle meningeal artery (MMA) was visualized, appearing dilated and hyperemic. **(C)** After bipolar electrocautery occlusion of the MMA, the vessel appeared pale and bloodless. **(D)** A one-month postoperative follow-up CT scan demonstrated near-complete resolution of the left chronic subdural hematoma. **(E)** Postoperative three-dimensional reconstruction revealed an enlarged bone flap measuring approximately 1.6 cm × 1.9 cm, with a residual bony opening of approximately 6 mm. The solid arrow indicates the patent MMA prior to occlusion; the dashed arrow indicates the occluded segment of the MMA post-procedure. S, Superior; P, Posterior; A, Anterior; I, Inferior; R, Right; L, Left.

The scalp was incised as planned to expose the skull. The surgical procedures in the observation group were shown in Figures 2A–E. A burr drill was used to create a bone hole with a diameter of approximately 6 mm at the incision point, which was determined through three-dimensional (3D) reconstruction (Figure 2A). Then, the skull was milled through the bone hole using a milling cutter to form a bone flap with a diameter not exceeding 2 cm. The MMA course was clearly visible beneath the bone flap. Bipolar electrocoagulation was used to burn the trajectory part of MMA within the bone window to ensure complete occlusion. After this, the MMA changed from a filled and expanded pale yellow color (Figure 2B) to a pale collapsed state without any blood (Figure 2C). Subsequently, a “+” shaped incision was made in the dura mater, allowing for the gradual release of the hematoma. After flushing the hematoma cavity with 37 °C saline until no bloody fluid flowed out, bipolar electrocoagulation was applied to cauterize the edges of the dural incision, forming a defect with a diameter of approximately 5 mm. A drainage tube was inserted through this channel at the lowest point of the subdural hematoma cavity, extending about 2.5 cm from the incision site through the bone hole, and connecting to a closed drainage system. After replacing and securing the bone flap, suturing and dressing were performed layer by layer. The postoperative 3D reconstruction revealed an enlarged bone flap measuring approximately 1.6 cm × 1.9 cm, with a residual bony opening of approximately 6 mm (Figure 2E). A follow-up CT scan 1 month after the procedure demonstrated nearly complete resolution of the left chronic subdural hematoma (Figure 2D).

In the control group, a cranial drill was used to drill a hole with a diameter of 1 cm, followed by an incision of the dura mater in a “+” shape. After the hematoma was gradually cleared, the hematoma cavity was flushed with 37 °C saline until clear fluid was observed. Bipolar electrocoagulation was then applied to cauterize the edges of the dural incision, retracting it to form a defect with a diameter of approximately 5 mm. A drainage tube was placed through this channel at the lowest point of the subdural hematoma cavity, extending about 2.5 cm from the incision site through the bone hole, and connecting to a closed drainage system. Suturing and dressing were then performed layer by layer.

**Figure 3:**
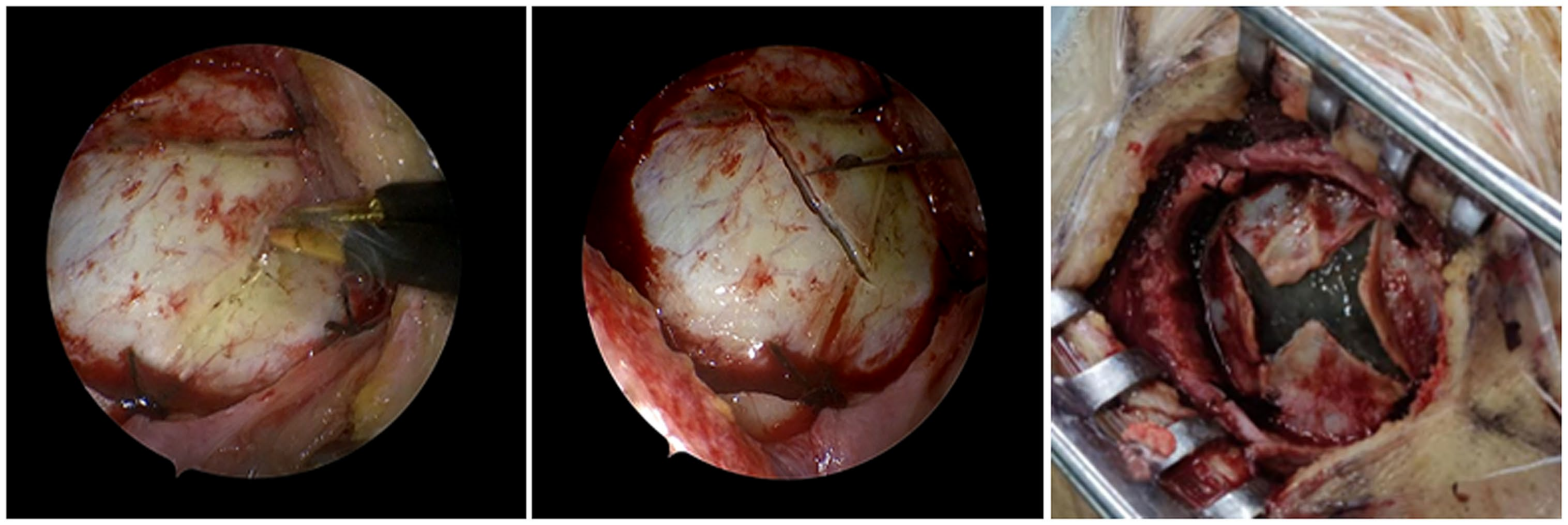
The verification of MMA occlusion.

During the operation, the MMA was cut when an incision was made on the dura mater. The MMA was checked to confirm that there was no bleeding at the severed end of the MMA, as shown in Figure 3. Then, the MMA occlusion was verified.

### Postoperative management

All patients received consistent treatment regimen in the neurosurgery ward after surgery, in addition to treating their underlying diseases. The regimen included oral administration of atorvastatin calcium at a dose of 20 mg per day, drainage system positioned about 8 cm below the interventricular foramen level, and removal of the drainage tube on the 5th day after surgery or when the drainage volume was less than 30 mL within 24 h. No hormone therapy was used in the patients.

### Data collection and follow-up

Data were collected by two senior neurosurgeons, and in case of disagreement, consensus was reached after discussion. Head CT or MRI scans were performed within 24 h, 1 month, 3 months, and 6 months after surgery, or whenever the patient experienced worsened symptoms such as headache, nausea, vomiting, limb numbness, or epilepsy. Observation indicators included gender, age, smoking history, drinking history, hypertension, diabetes, history of oral antithrombotic drugs (antiplatelet drugs and anticoagulants), use of statins, modified Rankin Scale (mRS) score at admission, hematoma thickness, degree of midline shift, duration of surgery, intraoperative blood loss, hematoma clearance rate, duration of drainage tube placement, length of hospital stay, hospitalization costs, perioperative complications, residual hematoma volume 1 month after surgery, and whether hematoma recurrence occurred within 6 months after surgery.

Hematoma recurrence was confirmed by head CT or MR examination. Hematoma recurrence was defined as the recurrence of a subdural fluidic hematoma on the operated side within 6 months after surgery, with a thickness exceeding 50% of the preoperative hematoma thickness, or causing midline displacement of at least half of the preoperative value. Hematoma recurrence was accompanied by the symptoms such as paralysis, drowsiness, intracranial hypertension, headache, nausea, vomiting, limb numbness, and epilepsy, which did not improve after medical treatment. Hematoma recurrence needed a second operation. The hematoma volume before reoperation was recorded to be the residual hematoma volume at 1 month after the first surgery.

Data collected at 6 months after surgery were referenced to the time of the first surgery. Perioperative complications included intraoperative brain parenchyma injury, postoperative acute subdural hematoma, infections (intracranial, incision or systemic), and thrombosis, but did not include hematoma recurrence.

Hematoma clearance rate was calculated as follows: maximum thickness of hematoma before surgery—maximum thickness of hematoma on the first CT scan within 24 h after surgery / maximum thickness of hematoma before surgery * 100%. The residual hematoma volume at 1 month after surgery was determined as follows: maximum thickness of hematoma at 1 month after surgery / maximum thickness of hematoma before surgery * 100%. The mRS scores of patients before surgery and 6 months after surgery were recorded, and categorized into good prognosis (0–2 scores) and poor prognosis (3–5 scores).

CT scanning used the following settings for image review: GE LightSpeed VCT 64 row spiral CT machine; Scanning layer thickness at 5 mm; scanning conditions was set at 120kVp, 200 mA; brain window of width 80 HU and level 40 HU; bone windowof width 2000–4,000 HU and level 400–700 HU. MR scanning was performed using Siemens Magnetom Skyra 1.5 T MRI System, with a scanning sequence of T1 weighted imaging (T1WI), T2 weighted imaging (T2WI), and fluid attenuated inversion recovery sequence (FLAIR), at a scanning layer thickness of 1 mm.

### Statistical methods

Data analysis was conducted using IBM SPSS Statistics 22.0 software. The Shapiro–Wilk test was first used to assess normality of continuous variables. Those variables conforming to a normal distribution were expressed as mean ± standard deviation, and independent sample t-tests were used for inter-group comparisons. Variables that did not follow a normal distribution were described as median, and non-parametric Mann–Whitney U tests were used for inter-group comparisons. Categorical variables were expressed as number of cases (percentage), and chi-square tests or Fisher’s exact tests were used for inter-group comparisons. Cohen’s Kappa coefficient was used to assess the consistency of the hematoma recurrence assessments within 6 months postoperatively by two observers. The strength of the Kappa value was interpreted according to Landis and Koch’s criteria: *κ* ≤ 0.20 indicates slight agreement; 0.21–0.40 indicates moderate agreement; 0.41–0.60 indicates moderate agreement; 0.61–0.80 indicates high agreement; 0.81–1.00 indicates almost perfect agreement. All hypothesis tests were two-tailed, and a *p*-value < 0.05 was considered statistically significant.

## Results

### Baseline data

This study included a total of 116 patients, with 84 males (72.4%) and 32 females (27.6%), resulting in a male-to-female ratio of approximately 2.6:1. The average age was 68.50 ± 9.08 years, with ages ranging from 48 to 91 years. There were no statistically significant differences in demographic characteristics, past medical history, mRS scores at admission, hematoma thickness, or midline shift degree between the two groups at baseline (Table 1).

**Table 1:** Comparison of baseline characteristics between the observation and control groups.

Characteristic		Observation group(*n* = 41)	Control group(*n* = 75)	*χ^2^*-value/*t*-value	*p* value
Gender	Male	29(70.7%)	55(73.3%)	0.090	0.764
Age	years	70.29 ± 10.38	67.52 ± 8.20	1.582	0.116
Smoking history	Yes	21(51.2%)	41(54.7%)	0.127	0.846
Alcoholism consumption	Yes	17(41.5%)	37(49.3%)	0.660	0.442
Hypertension	Yes	16(39.0%)	25(33.3%)	0.376	0.540
Diabetes mellitus	Yes	6(14.6%)	13(17.3%)	0.141	0.707
Maximum preoperative hematoma thickness	cm	2.70 ± 0.42	2.57 ± 0.41	1.550	0.124
Maximum preoperative midline shift distance	cm	1.23 ± 0.35	1.16 ± 0.40	0.992	0.323
mRS on admission	good	34(82.9%)	60(80.0%)	0.701	0.807

### Perioperative and follow-up outcomes

There were no statistically significant differences between the two groups in terms of operation duration, intraoperative blood loss, hematoma removal effect, perioperative complications, hospitalization costs, or the use of statins and antithrombotic drugs. The mRS scores at 1 month or 6 months postoperatively were also comparable in both groups. However, patients in the observation group had shorter drainage tube placement duration, shorter hospitalization duration, and reduced residual hematoma volume at 1 month post-operation compared to those in the control group (*p* < 0.001; Table 2). The hematoma recurrence rate at 6 months post-operation was significantly lower in the observation group (2/41, 4.9%) than in the control group (15/75, 20.0%; *p* = 0.030; Table 2). In the observation group, 41 patients underwent 3D reconstruction technology to locate the MMA, which was identified in all enlarged bony foramen. The consistency of the assessment results between two observers on hematoma recurrence within 6 months postoperatively was almost perfect (*κ* = 0.82, 95% CI: 0.67 to 0.97, *p* < 0.001).

**Table 2:** Comparison of perioperative outcomes and follow-up results between the observation and control groups.

Intraoperative and postoperative outcomes	Observation group (*n* = 41)	Control group (*n* = 75)	*χ^2^*-value/*t*-value	*p* value
Operative time (min)	42.22 ± 6.08	41.05 ± 6.20	0.976	0.331
Intraoperative blood loss (ml)	23.41 ± 8.33	20.47 ± 8.55	1.792	0.076
Hematoma evacuation efficacy (%)	86.44 ± 7.77	84.97 ± 9.81	0.825	0.411
Duration of drainage (days)	2.15 ± 0.69	3.04 ± 0.73	6.451	<0.001
Antithrombotic use	13(31.7%)	19(25.3%)	0.463	0.517
Statins use	32(84.2%)	40(88.9%)	0.392	0.531
Perioperative complications	1(2.4%)	7(9.3%)	1.962	0.257
Length of hospital stay (days)	8.90 ± 1.48	9.97 ± 1.38	3.903	<0.001
Total hospitalization costs (RMB, Yuan)	9875.56 ± 716.22	9664.39 ± 547.42	1.777	0.078
Residual hematoma at 1 month postoperatively	22.37 ± 14.33	35.68 ± 16.62	4.324	<0.001
Hematoma recurrence within 1 month postoperatively	0(0%)	4(5.3%)	Fisher	0.296
mRS at 6 months postoperatively	38(92.7%)	68(90.7%)	0.137	0.711
Hematoma recurrence within 6 months postoperatively	2(4.9%)	15(20.0%)	4.847	0.030

## Discussion

This study proposes a surgical approach that utilizes 3D reconstruction technology to simultaneously drain the hematoma and occlude the MMA through the same incision in a single procedure. This approach not only resolved the hematoma occupying effect with a shorter drainage tube placement duration and hospitalization duration, but also reduced residual hematoma volume and the risk of postoperative hematoma recurrence in CSDH patients.

In this study, it was found that the recurrence rate in the control group was 20.0%, which is similar to the reported recurrence rate (22.5%) of hematoma after BHD by Atefi et al. (17), but higher than that (13.4%) reported by Carter et al. (18). The recurrence rate in the observation group was approximately 4.9%, which exhibited a statistically significant difference compared to the control group, and much lower than that in the previous reported cohort (17, 18). This difference may be attributed to the following two factors.

On the one hand, patients in the observation group underwent bipolar coagulation during surgery to block the MMA on the dura mater, which cut off the blood supply to the hematoma cavity and reduced the source of fluid in the hematoma cavity. Continuous leakage from newly formed capillaries in the hematoma capsule is an important factor in the formation and postoperative recurrence of CSDH, and the blood supply to these newly formed capillaries originates from the MMA (19, 20). Therefore, the MMAE can prevent hematoma progression and reduce postoperative recurrence. On the other hand, the 3D reconstruction displayed the location of the hematoma in the skull and the pressure trace of the MMA on the skull using patient’s cranial CT data in a very clear way. Some studies (11, 21) have found that the main trunk and its main branches of the MMA run along the lateral surface of the dura mater, closely adhering to the skull and leaving a pressure trace on the skull. CSDH is mainly located in the anterior region and is supplied by the anterior branches of the MMA. Retaining the anterior branches of the MMA during craniotomy is a risk factor for postoperative CSDH. In this study, 3D reconstruction was performed using patient’s cranial CT data, the pressure trace of the MMA on the inner table of the skull within the hematoma area was identified. Surface localization was carried out with reference to the patient’s own bony structure. The surgical approach was performed above the thicker hematoma area and coincide with the MMA trajectory. To ensure effective hematoma removal, the MMA was blocked during the procedure. Efforts were made to block the proximal end of the main trunk of the MMA branches as thoroughly as possible to achieve greater dearterialization of the dura mater. Research (22) shows blocking the anterior and posterior branches of the MMA may increase hematoma absorption, but there is not enough evidence for the effectiveness of blocking both branches on postoperative hematoma recurrence. Additionally, most of these patients in the current study were elderly with poor surgical tolerance, and the hematoma within the hematoma cavity has been completely removed during surgery. Therefore, in this study, the observation group blocked the anterior branch or the main trunk of the MMA through surgical intervention, without performing more invasive surgery to block additional MMA branches.

MMA occlusion in surgery avoids some MMAE deficiencies. MMAE cannot immediately relieve hematoma pressure, making it unsuitable for patients with severe discomfort (23). MMAE also requires contrast agents, which some patients cannot tolerate (24). Additionally, MMAE poses risks due to abnormal anastomosis and requires specialized equipment and training (25, 26). It also involves radiation exposure and high medical costs, limiting its application (4, 12).

This study used a new method (3D reconstruction) to locate the MMA while treating CSDH before the operation (11–13, 27). Previous reports use a head positioning template to mark the positioning incision, which requires specialized positioning equipment, and may not be suitable to all patients due to anatomical differences among individuals (11). Using navigation equipment or radiographic fluoroscopy to locate MMA during surgery can enhance the precision of the approach, but specialized equipment and technical personnel limits the application in medical institutions with less developed economic levels (11, 12). The extra waiting time for 3D printing technology to position MMA makes it difficult to apply this technology for emergency patients (13).

Patients in the observation group had expanded drilling during surgery. This allowed for safer and more accurate location of the MMA. The bone hole was also closer to the forehead. The superficial layer of the bone flap contains periosteum, galea aponeurotica, and temporal muscle. Residual fluid may flow through the cut dura mater, bone hole, and bone sutures to the outside of the bone flap and communicate with these soft tissues. This not only increases the distribution range of residual fluid, serving as subperiosteal drainage, but also suggests that the periosteum, galea aponeurotica, and temporal muscle may play a role in absorbing residual fluid. The mechanism is speculated to be similar to that of temporal muscle application in the treatment of subdural effusion (27, 28). This absorption may be why the observation group had shorter drainage tube use, hospital stays, and less residual hematoma after a month. Due to the short drainage tube indwelling time and rapid hematoma absorption, the patients’ hospitalization duration was also shorter. After removing the drainage tube, the residual fluid in the hematoma cavity could still be absorbed through the temporal muscle and galea aponeurotica, resulting in a smaller residual hematoma volume at 1 month after surgery. Compared with the control group undergoing traditional trepanation and drainage, this surgical procedure is simple to perform, causes minimal trauma, does not affect the hematoma removal effect, and does not increase medical economic expenditure, while it achieved similar results to the control group in terms of operation duration, intraoperative blood loss, hematoma removal effect, perioperative complications, and hospitalization costs.

According to previous reports, a bone hole with a diameter of approximately 14 mm is typically drilled in the skull during BHD (14). Postoperatively, patients may often fell embarrassed and troubled due to local scalp depression caused by soft tissue atrophy near the bone hole (29, 30). Up to 64% of patients may experience limitations in activities of daily living due to cosmetic concerns (29). Although patients in the observation group underwent expanded drilling, the bone flap was reset and fixed postoperatively, leaving only a bone hole with a diameter of approximately 6 mm for hematoma drainage. This aimed to reduce the occurrence of such complications.

It should be noted that there was no statistically significant difference in mRS scores at 6 months after surgery, indicating that this surgical procedure did not improve patients’ neurological recovery at 6 months postoperatively. In addition, this study found that 72.4% of the patients were male, which was a significantly higher proportion more than females. Among them, the proportion of male patients was 70.7% in the observation group, and 73.3% in the control group, without statistically significant difference. This finding is consistent with previous reports, which also find most of CSDH patients are males (1, 31, 32).

This study also has some limitations. Firstly, this is a single-center retrospective study where patients were grouped based on their actual surgical plans. We screened patients based on the electronic medical record system, and excluded those who received antithrombotic therapy within 1 month after surgery, were lost to follow-up, or had incomplete data. This study design may lead to a small sample size, an unbalanced sample size between groups, and patient selection bias, thus affect the results. Secondly, the method of locating MMA relied on the impression of MMA on the skull, which may not be applicable to patients with moyamoya disease, skull lesions, or those who had a craniotomy. Thirdly, this study determined the local occlusion of MMA under direct vision during surgery, without performing specialized MMA imaging examinations before and after surgery to directly show the distribution of MMA and the extent of MMA occlusion. The lack of angiographic confirmation may influence the results. Fourthly, this study only included patients with unilateral CSDH who underwent initial surgical treatment, and further research is needed to determine whether the findings apply to patients with bilateral CSDH or those with postoperative hematoma recurrence. Fifthly, the results may be influenced by the proficiency of the surgeons, patients’ lifestyles, etc., which were not analyzed in this study. These limitations restrict the generalizability of this study. More research with large, multi-center data is needed to confirm the results. A comparison with MMAE combined with BHD is needed to clarify its superiority or inferiority in reducing postoperative hematoma recurrence.

## Conclusion

This improved surgical technique performed BHD and MMA through a single point under 3D reconstruction technology. This improved procedure retains the benefits of minimal invasion and immediate hematoma clearance fronm the traditional procedure. In addition, it reduces hospitalization stay, duration of drainage tube placement, and postoperative hematoma recurrence without increasing surgical risk, difficulty and hospitalization costs. Further verification with large-scale, multi-center prospective data and long-term follow up is needed.

## Data Availability

The original contributions presented in the study are included in the article/supplementary material, further inquiries can be directed to the corresponding author.
